# Impact of the Preservation of Residual Kidney Function on Hemodialysis Survival: Results from the BISTRO Trial

**DOI:** 10.34067/KID.0000000596

**Published:** 2024-10-10

**Authors:** John Belcher, David Coyle, Elizabeth J. Lindley, David Keane, Fergus J. Caskey, Indranil Dasgupta, Andrew Davenport, Ken Farrington, Sandip Mitra, Paula Ormandy, Martin Wilkie, Jamie Macdonald, Ivonne Solis-Trapala, Julius Sim, Simon J. Davies

**Affiliations:** 1 School of Medicine, Keele University, Keele, United Kingdom; 2 NIHR Devices for Dignity, Sheffield Teaching Hospitals NHS Foundation Trust, Sheffield, United Kingdom; 3 Renal Medicine, Leeds Teaching Hospitals NHS Trust, Leeds, United Kingdom; 4 CÚRAM SFI Research Centre for Medical Devices, University of Galway, Galway, Ireland; 5 Population Health Sciences, University of Bristol, University of Bristol, Bristol, United Kingdom; 6 Renal Medicine, University Hospitals Birmingham NHS Foundation Trust, Birmingham, United Kingdom; 7 UCL Department of Renal Medicine, Royal Free Hampstead NHS Trust, University College, London, United Kingdom; 8 Renal Medicine, East & North Hertfordshire NHS Trust, Stevenage, United Kingdom; 9 Manchester Academic Health Sciences Centre (MAHSC), University Hospital Manchester, Manchester, United Kingdom; 10 School of Health and Society, University of Salford, Manchester, United Kingdom; 11 Renal Medicine, Sheffield Teaching Hospitals NHS Foundation Trust, Sheffield, United Kingdom; 12 Institute of Applied Human Physiology, Bangor University, Bangor, United Kingdom

**Keywords:** chronic hemodialysis, clinical trial, ethnicity, mortality risk

## Abstract

**Key Points:**

- Residual kidney function during the first 2 years of hemodialysis treatment is associated with a long-term (>4 years) survival advantage.
- Incorporating bioimpedance measurements to inform the setting of the postdialysis target weight does not affect patient survival.

**Background:**

Preservation of residual kidney function (RKF) in dialysis patients has been associated with improved survival. RKF in the BISTRO trial was relatively well preserved, and in this study, we describe its association with survival during the trial and extended follow-up.

**Methods:**

RKF, measured as the average urea and creatinine clearance (GFR) or 24-hour urine volume, was assessed at baseline; 1, 2, and 3 months; and every three months for up to 2 years in incident hemodialysis patients. Time to event survival data or competing events (transplantation, modality change) was obtained for 50 months after enrollment *via* data linkage with the UK Renal Registry. Cox proportional hazards regression survival models, including those incorporating change in GFR from baseline as a time-varying variable and joint regression models for longitudinal and survival data (longitudinal models for GFR or urine volume), were used to explore the relationship of RKF preservation with survival. Analyses were adjusted for age, sex, comorbidity, and ethnicity.

**Results:**

A total of 2919 measures of RKF were made in 387 patients from 32 UK dialysis units. Higher age and comorbidity score were associated with increased mortality in all models. Baseline GFR reduced the risk of death (hazard ratio [HR], 0.918; 95% confidence interval [CI], 0.844 to 0.999) per ml/min per 1.73 m^2^. A greater fall in GFR and urine volume from baseline was associated with a nonsignificant increased risk of death, as visualized on spline plots. In the joint survival models, higher GFR (adjusted HR, 0.88; 95% CI, 0.80 to 0.97) or urine volume (adjusted HR, 0.75, 95% CI, 0.57 to 0.95/L) at any time point was associated with better survival.

**Conclusions:**

Lower RKF during the first 2 years of hemodialysis is associated with an increased death risk for up to 50 months after dialysis initiation. This adds to a growing body of evidence that interventions to preserve RKF should be developed and tested in clinical trials.

## Visual Abstract

**Figure:**
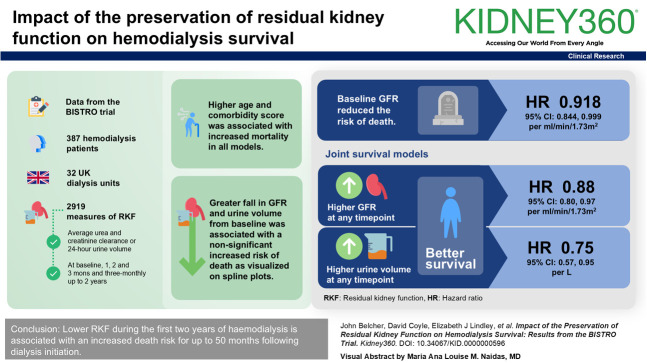

## Introduction

Residual kidney function (RKF) has been associated with better survival for both hemodialysis and peritoneal dialysis patients.^1–4^ However, data supporting this assertion are less plentiful for hemodialysis than peritoneal dialysis, most likely because RKF, typically expressed either as the measured GFR (GFR, *e.g*., average of the urinary urea and creatinine clearance) or as the urine volume, is not commonly measured in the hemodialysis units. The Netherlands Cooperative Study on the Adequacy of Dialysis was the first large study to demonstrate a positive association between GFR and survival for hemodialysis patients.^1^ Treating GFR as a time-varying continuous explanatory variable in a Cox proportional hazards regression model, they reported that greater GFR was associated with better survival at any time on dialysis over and above the effects of any observed variation in dialysis-delivered urea clearance. It was not clear from this analysis whether the survival benefit was primarily driven by GFR at the start of dialysis or the subsequent individual change in GFR. More recently, Obi *et al.* found that the mortality rate up to 4 years of dialysis was proportional to the rate of fall in GFR during the first year of treatment, determined from just two measurements, taken first at baseline and then again at 12 months.^2^ This would suggest that it is loss of RKF over time, rather than the amount of RKF at the start of dialysis, that is more important, a strong argument for developing strategies aiming to preserve RKF over time. By design, patients in this study^2^ had to survive at least 1 year on dialysis, potentially introducing bias given that these early deaths will have occurred when the loss in RKF is fastest, and although the association of mortality with decline in RKF was observed for each category of baseline GFR, the relative importance of the baseline measurement is not completely clear.

The BISTRO trial was designed to establish if by avoiding excessive fluid removal during dialysis, RKF could be better preserved, using bioimpedance as an aid to the process of setting the postdialysis target weight.^5,6^ A prespecified secondary analysis of the trial was to determine whether the preservation in RKF during the first 2 years of treatment (or any part thereof) was associated with subsequent survival after extended follow-up of the trial cohort. The particular value of this analysis includes frequent measurement of RKF as determined from the mean of the urea and creatinine clearance using a validated calculator over a 2-year period,^7^ from which a more precise estimate of the decline in RKF (GFR and urine volume) could be made when compared with previous studies and with up to 5-year follow-up. Given the limitations of the methods described above, combined with the difficulty in defining a run-in baseline period in this incident dialysis population, during which mortality events will have likely occurred, we undertook a combination of analyses to establish whether there is a link between RKF and survival in the BISTRO trial cohort. In addition to a baseline Cox proportional hazards survival regression model, in which the first measurement of RKF was used, we undertook a modeling incorporating the change in RKF from baseline as an additional time-varying covariate, visualizing the relationship of the change with the hazard of death using spline plots. Finally, we developed a joint model comprising of longitudinal and survival models, the former including the measurement if RKF (GFR and urine volume) at each point in time.

## Methods

**Figure 1:**
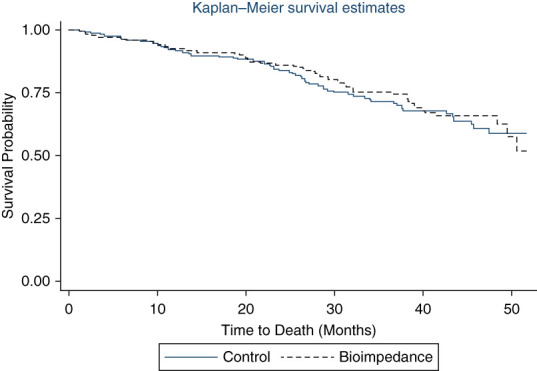
Kaplan–Meier survival plot of the BISTRO trial including the extended period of follow-up beyond trial completion by group randomized to control and intervention (bioimpedance).

BISTRO was a pragmatic, multicenter (32 UK dialysis centers), randomized controlled trial conducted in 439 incident hemodialysis patients with a urine output >500 ml/d or a GFR >3 ml/min per 1.73 m^2^.^5,6^ GFR, indexed to BSA using the Dubois formula,^8^ was measured using a validated calculator that enables the use of estimated blood results if actual samples are unavailable to calculate the mean urea and creatinine clearance from interdialytic urine collections taken monthly for 3 months and then every 3 months for up to 24 months or until study dropout. Investigators had an excel spreadsheet that calculates the GFR for patients undergoing one, two, or three dialysis sessions per week based on a steady-state model that includes postdialysis solute rebound. Audit of 316 GFR measures found that the steady-state assumption held true for >97%. RKF could also be expressed as the interdialytic urine volume normalized to 24 hours (collection periods ranged between 24 and 48 hours).^7^ For this analysis, we pooled data from the trial arms because there were no between-group differences in either the rate of RKF decline (GFR or urine volume), reasons for dropout, or how close the set postdialysis target weight was to the bioimpedance derived normally hydrated weight (difference in mixed-effects model adjusted for age, sex, comorbidity, and current RKF being 0.11 kg; 95% confidence interval [CI], −0.282 to 0.498). Furthermore, as shown in Figure 1, there was no difference between the trial arms in survival over the 50 months of follow-up.

To obtain data on patient outcomes after completion of the trial, participant data were linked to the time-line information collected by the UK Renal Registry (UKRR). All patients had given their written consent before inclusion in the trial which specifically included permissions for this data linkage, which was underpinned by a data sharing agreement approved by the trial sponsor (Keele University), and ethical approvals were obtained through the UK Integrated Research Application System (Project number 20613). For this analysis, we used ethnicity data collected by the UKRR because the data collected using the clinical reporting forms during the trial allocated a large number of participants as ‘other’ without providing further information.^6^

### Statistical Analysis

Patients censored in the main trial had their transplantation and survival follow-up outcomes updated from the UKRR up to 50 months from randomization. Patients who received a transplant were censored at their transplant date, and deaths were identified as new events. For those not experiencing either transplantation or death, event times were censored using the latest recorded urea reduction ratio or hemodialysis session or the last date recorded by the registry up until December 31, 2021. Three approaches to analyzing the relationship between RKF and survival on dialysis were used. The first was a Cox proportional hazards regression survival model incorporating the baseline measurement of GFR (defined here as the first measure obtained within 3 months of randomization) to model the time to event (all-cause mortality) with *a priori* selected explanatory variables, chosen as the key determinants of survival, including age, sex, ethnicity, and comorbidity, with the latter using the externally validated Stoke Comorbidity Score, which encompasses primary kidney diseases associated with increased mortality, notably diabetes and renovascular disease.^4,9^ The second approach was a Cox proportional hazards regression incorporating changes from baseline in GFR or urine volume as a time-varying covariate, with measurements below baseline expressed as negative values, using the last-observation carried-forward approach as described by Arisdo *et al.*^10^ Restricted cubic spline terms with 5 degrees of freedom were introduced to capture any potential nonlinearities between changes in GFR or urine volume and mortality risk. Knots were place at points recommended by Harrell.^11^ The proportional hazards assumption was checked using the Schoenfield residual test for individual covariates and a global test using the *stata phest* command in STATA. The third approach was the construction of a joint model in which the longitudinal component modeled the trajectory of the GFR or urine volume using a linear mixed-effects model with random slope and intercept, thereby accounting for the nonindependence of repeated measures and allowing for irregular spacing of sample collections, biological variance, and measurement error. This approach allowed us to incorporate all the measurements, thus improving the efficiency of the model and reducing bias, for example, with respect to informative censoring. To accommodate the nonlinear decline in RKF a quadratic trajectory was considered for the longitudinal model. Analyses were undertaken using Stata and the *JMBayes2*^12^ and *Survival*^13,14^ packages in R.^15^

## Results

**Figure 2:**
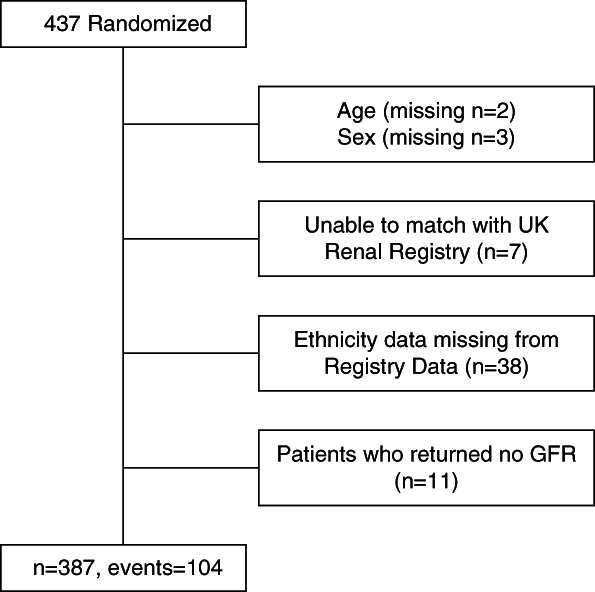
Study flow diagram.

Four hundred thirty-nine patients were randomized in the trial, which ran between April 2017 and October 2019 (minimum within trial follow-up 12 months, maximum 24 months, extended follow-up until end of 2021). Figure 2 shows that the final number included in the analyses with complete case data was 387, and their baseline demographics are shown in comparison with all trial participants in Table 1. The analyses are based on 2919 measurements of RKF.

**Table 1:** Baseline characteristics of the overall trial participants and those included in the analyses (see Figure 2)

Characteristic	Overall Trial Participants (*N*=437)	Trial Participants Included in the Survival Analyses (*n*=387)
Sex; male/female, *No.* (%)	306 (30.0)/128 (70.0)	266 (31.3)/121 (68.7)
Hemodialysis modality; hemodialysis/HDF, *No.* (%)	295 (67.5)/140 (32.0)	269 (69.5)/118 (30.5)
Age, mean (SD)	61.34 (14.11)	61.29 (14.01)
**Ethnicities**		
Asian	44 (10.1)	43 (11.1)
Black	37 (8.5)	37 (9.6)
Missing	42 (9.6)	—
Mixed	3 (0.7)	3 (0.78)
Other	1 (0.2)	0 (0)
White	310 (70.1)	304 (78.6)
Planned/unplanned start, *No.* (%)	364 (83.3)/71 (16.2)	324 (83.7)/63 (16.3)
Access; fistula/graft/line, *No.* (%)	231 (52.9)/7 (1.6)/197 (45.1)	210 (54.3)/6 (1.6)/171 (44.2)
Years since primary diagnosis, median (IQR)	4.02 (1.02–9.13)	4.06 (1.11–9.27)
**Comorbidities, *No.* (%)**		
Malignancy	28 (6.4)	26 (6.7)
Ischemic heart disease	88 (20.1)	77 (19.9)
Peripheral vascular disease	52 (11.9)	44 (11.4)
Left ventricular dysfunction	56 (12.8)	51 (13.2)
Diabetes mellitus	198 (45.3)	177 (45.7)
Systemic collagen vascular disease	13 (2.9)	10 (2.6)
Comorbidity score, median (IQR)	1 (0–2)	1 (0–2)
Measured GFR^a^ (ml/min per 1.73 m^2^), median (IQR)	4.27 (2.92–6.05)	4.38 (3.01–6.13)
Urine volume, L, median (IQR)	1.48 (0.94–2.28)	1.50 (0.95–2.3)
Deaths	121	104
No. of baseline measures of RKF by month=3		423

HDF, hemodiafiltration; IQR, interquartile range; RKF, residual kidney function.

a GFR=residual kidney function measured as GFR.

**Figure 3:**
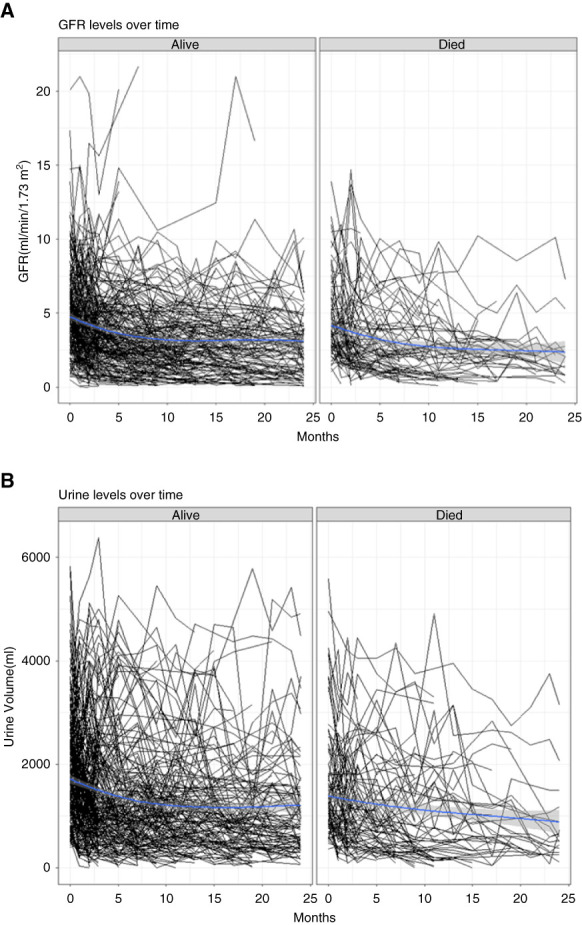
**Longitudinal changes in RKF during the trial, illustrating the large within and between patient variation.** Spaghetti plots showing the individual changes in (A) GFR and (B) urine volume during the trial follow-up according to whether participants were alive (left panel) or dead (right panel) following extended follow-up (up to 50 months).

**Figure 4:**
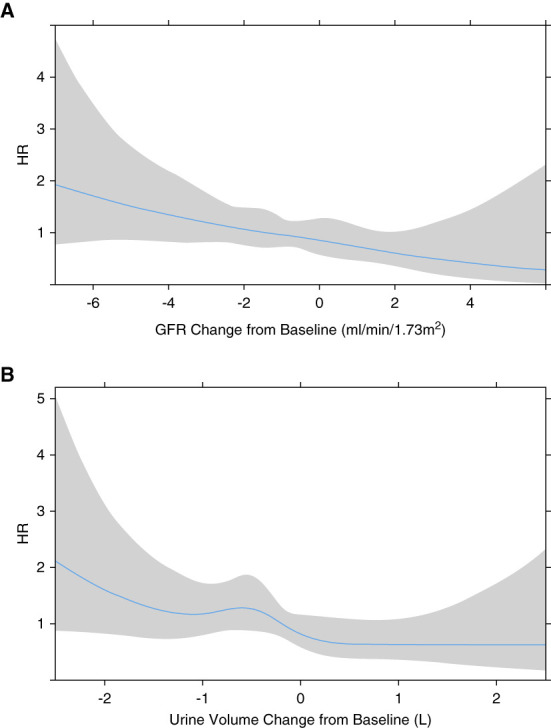
**A greater decline in RKF from baseline is associated with a higher risk of death.** Spline plots illustrating the HR for mortality as a function of (A) the change in GFR (per ml/min per 1.73 m^2^ per month) and (B) the change in urine volume (ml) from baseline. HR, hazard ratio.

In all the survival models, increased risk of death was associated with increasing age and comorbidity score. Sex and ethnicity had no significant effects, although the hazard rate for White patients, compared with other ethnicities, tended to be higher. In the survival model incorporating the measured GFR at baseline (see Table 2), GFR was associated with a significant reduction in the risk of death hazard ratio, 0.918 per ml/min per 1.73 m^2^ (95% CI, 0.844 to 0.999). Spaghetti plots of the measured GFR or urine volume according to subsequent survival outcome are shown in Figure 3, illustrating the substantial within- and between-patient variation. In the Cox proportional hazards model incorporating the change from baseline in GFR or urine volume (adjusted for baseline values), neither measure of RKF was significantly associated with survival (Table 3). However, the spline plots of survival hazard and change in GFR and urine volume (Figure 4, A and B) demonstrated greater changes led to an increased risk of death. The joint survival models for GFR and urine volume are shown in Table 4. They demonstrate that for both measures of RKF at any time point during follow-up, especially when adjusted for baseline covariates, the current value of GFR or urine volume was associated with a reduction in the risk of death. The parameters for the quadratic polynomial for the longitudinal submodel were all significant and yielded a reasonable approximation of the data with a conditional *r*^2^ value of 80%.

**Table 2:** Estimated hazards ratios of mortality using a Cox proportional hazards regression model including baseline GFR

Covariate	HR	Lower 95% CL	Upper 95% CL	*P* Value
Age (per year)	1.046	1.028	1.065	<0.001
Sex (ref female)	0.971	0.634	1.487	0.892
Comorbidity score (per item)	1.348	1.148	1.584	<0.001
**Ethnicity (South)**				
Asian	—	—	—	—
Black	1.263	0.455	3.509	0.654
Mixed	<0.00	0.000	∞	0.995
Other	Not computable	Not computable	Not computable	Not computable
White	1.800	0.832	3.905	0.135
Baseline GFR^a^ (within 3 mo)	0.918	0.844	0.999	0.046

CL, confidence limits; HR, hazard ratio.

a Unit for GFR hazard ratio=per ml/min per 1.73 m^2^.

**Table 3:** Hazard ratios for time-varying Cox models for GFR and urine volume

Covariate	GFR		Urine Volume	
	HR (95% CI)	*P* Value	HR (95% CI)	*P* Value
Baseline RKF	0.89 (0.79–1.01)	0.079	0.81 (0.62–1.05)	0.104
Change in RKF from baseline	0.94 (0.83–1.06)	0.296	1.02 (0.77–1.07)	0.869
Age (per year)	1.05 (0.95–1.03)	<0.001	1.05 (1.03–1.07)	<0.001
Sex: female	Reference		Reference	
Sex: male	0.97 (0.61–1.57)	0.920	0.98 (0.63–1.52)	0.929
Comorbidity (per item)	1.27 (1.07–1.51)	0.006	1.28 (1.09–1.50)	0.002
**Ethnicity**				
Asian	Reference		Reference	
Black	0.96 (0.31–3.06)	0.951	1.06 (0.39–3.72)	0.910
Mixed	Not computable	—	Not computable	—
White	1.62 (0.69–3.82)	0.266	1.41 (0.64–3.08)	0.391

Unit for GFR hazard ratio=per ml/min per 1.73 m^2^, units for urine volume hazard ratio=per L/d. HR, hazard ratio; CI, confidence interval; RKF, residual kidney function.

**Table 4:** Estimated joint models of survival and longitudinal residual kidney function measurements (model 1: GFR, model 2: urine volume)

Survival Model	GFR		Urine Volume	
	HR (95% CI)	*P* Value	HR (95% CI)	*P* Value
**Association**				
RKF^a^	0.88 (0.80 to 0.97)	0.006	0.75 (0.57 to 0.95)	0.014
**Unadjusted**				
RKF^a^	0.91 (0.82 to 0.99)	0.048	0.80 (0.62 to 1.03)	0.079
Age (per year)	1.05 (1.03 to 1.07)	<0.001	1.05 (1.03 to 1.07)	<0.001
Sex: female	Reference		Reference	
Sex: male	1.02 (0.66 to 1.54)	0.909	0.99 (0.65 to 1.51)	0.984
Comorbidity	1.31 (1.11 to 1.53)	0.003	1.32 (1.13 to 1.54)	<0.001
**Ethnicity**				
Asian	Reference		Reference	
Black	1.13 (0.33 to 3.72)	0.825	1.22 (0.38 to 3.88)	0.747
Mixed	Not computable		Not computable	
White	1.63 (0.79 to 3.88)	0.210	1.58 (0.77 to 3.81)	0.248

Longitudinal Model	Coefficient (SEM)	*P* Value	Coefficient (SEM)	*P* Value
Intercept	4.577 (0.136)	<0.001	1.626 (0.048)	<0.001
Slope	−0.208 (0.014)	<0.001	−0.062 (0.005)	<0.001
Quadratic	0.004 (0.001)	<0.001	0.001 (0.0002)	<0.001

CI, confidence interval; HR, hazard ratio; RKF, residual kidney function.

a Hazard ratio of all parameters reported as ratio of hazard rates of all-cause mortality per one unit increase or for a category with respect to a reference group, at any point in time, units for GFR hazard ratio=per ml/min per 1.73 m^2^, for urine volume=per L/d.

## Discussion

This secondary analysis of the BISTRO trial provides further evidence that RKF is associated with a survival benefit for hemodialysis patients for up to 5 years of treatment.^1,2,16–18^ This emphasizes the relative importance of the relative value of RKF maintained over time versus the absolute amount of RKF at the start of treatment, using more granular data than in the study by Obi *et al.,*^2^ and includes patients from within 3 months of starting treatment, reflecting the inclusion criteria for the trial.^5^ Our study also provides further evidence of the value of making regular assessments of RKF in hemodialysis patients, especially because it is associated with other benefits, including reduced hospital admissions, reduced depression, and better quality of life,^16,19,20^ whether it is measured as GFR or urine volume. As such, it highlights the need for investigation of clinical practices, including incremental dialysis,^21,22^ and therapeutic strategies, such as SGLT2 inhibitors, that may have the potential to reduce the rate of decline of RKF. This analysis also found no effect of the availability of bioimpedance readings during the first 2 years of dialysis treatment on subsequent survival. Given that the BISTRO trial found that the difference between the bioimpedance-derived normally hydrated weight and the target weight, and the decline in RKF was not different by trial arm, this is not surprising.

One of the strengths of our analysis is that we have used complementary statistical methods that allow for inclusion of all of the BISTRO cohort with a full set of baseline data. Of the approaches considered, the Cox proportional hazards model incorporating change from baseline as a time-varying covariate offers more flexibility in capturing the relationship between GFR or urine volume and the hazard of death, and the spline plot yields a simple graphical summary for ease of interpretation while noting that the change from baseline in both the GFR and urine volume was not statistically significant. This likely reflects the fact that there is considerable within-patient variation in the longitudinal decline in RKF, as illustrated in Figure 3. Although having multiple longitudinal measures of RKF is a strength of this analysis, it also describes the reality of change in RKF over time, emphasizing the likely biological variation that occurs and possible inaccuracies in the measurement methods. As a result, the relationship with long-term survival has to be treated with caution.

The advantage of the joint model is that it allows informative censoring to be taken into account, in particular deaths during the first year of treatment, showing that the amount of RKF a patient has at any time point after the start of dialysis has survival value. However, we were only able to examine the effect of RKF during the first 2 years of treatment, meaning that the joint model makes assumptions as to the subsequent trajectory of RKF for those surviving beyond 2 years, although it seems likely that the trajectory of decline in kidney function is well established by this time. Our analysis of the primary outcome data indicated that time to anuria was linear over the 2 years of follow-up, with a more rapid decline in RKF occurring during the first year compared with the second year of treatment (year 1: −0.178 (95% CI, −0.196 to −0.159) and year 2: −0.061 (95% CI, −0.086 to −0.036) ml/min per 1.73 m^2^ per month). Data from other studies suggests that this subsequent, slower decline may persist for some years.^16^

This analysis has some limitations. Trial participants are not necessarily typical of the wider dialysis population and, in this case, may reflect a group that has relatively well-preserved RKF—especially as BISTRO was designed to avoid unnecessary volume depletion during dialysis sessions. However, the trial was deliberately pragmatic in design, and the characteristics of those recruited were not markedly different from the UK dialysis population as a whole.^6,23^ Trial participants did miss measures of RKF, partly because the trial was interrupted by the Coronavirus Disease 2019 pandemic, but despite this, our study reflects the most complete dataset published so far. For this analysis, we used the ethnicity data collected from the UKRR, rather than that from the trial records, because these were more complete. This analysis was not powered to look at different causes of death or survival by ethnicity, although the trend for higher mortality in White patients is in keeping with other published studies.^24^ Our cohort was relatively small, with just 104 deaths, limiting the number of covariates we could include in our survival models, and thus, we did not adjust for potential confounders, such as albumin and inflammation, although it can be argued that loss in RKF is one of the important determinants of these mediators of mortality in dialysis patients. We did not include an analysis of the determinants of the rate of decline in kidney function, of which there are many potential candidates, although in the primary trial analysis, we did not see a significant association with age, sex, cumulative comorbidity, or a number of medications prescribed at baseline, including diuretics, renin-angiotensin system blockade, or calcium antagonists. The modest association with ethnicity (faster decline in Asian patients) was opposite to the trend with mortality already discussed above. Despite these limitations, this extended follow-up of the BISTRO cohort provides further strong evidence of the importance of maintaining RKF.

In conclusion, this analysis of the BISTRO trial cohort adds further weight to the argument that preserving RKF in dialysis patients should be an important clinical and research goal. In addition to confirming the findings of previous studies, including the relative importance of urine volume, not just calculated GFR, it strongly suggests that the survival benefits are associated with both baseline and maintained RKF over time and that they are apparent for several years of treatment after dialysis is initiated. Survival was not affected by the availability of bioimpedance estimates of the normally hydrated weight when setting the postdialysis target weight.

## Supplementary Material

## Data Availability

Original data created for the study are or will be available in a persistent repository on publication. Raw Data/Source Data. Figshare: Lippincott Data Repository. Available on request from the corresponding author.
