# Supplementary material for: Impact of the Preservation of Residual Kidney Function on Hemodialysis Survival: Results from the BISTRO Trial
Source: Kidney360. 2024 Oct 10;6(1):112–20. doi: 10.34067/KID.0000000596 (PMC11793177; doi:10.34067/KID.0000000596)
Supplement: Supplementary file 1 [file kidney360-6-112-s001.pdf]

ASN Journal Disclosure Form

As per ASN journal policy, I have disclosed any financial relationships or commitments I have held in the past 36 months as included below. I have listed my Current Employer below to indicate there is a relationship requiring disclosure. If no relationship exists, my Current Employer is not listed.

J. Belcher reports the following:
Employer: Keele University

I understand that the information above will be published within the journal article, if accepted, and that failure to comply and/or to accurately and completely report the potential financial conflicts of interest could lead to the following: 1) Prior to publication, article rejection, or 2) Post-publication, sanctions ranging from, but not limited to, issuing a correction, reporting the inaccurate information to the authors' institution, banning authors from submitting work to ASN journals for varying lengths of time, and/or retraction of the published work.

Name: John Belcher
Manuscript ID: K360-2024-000151R2
Manuscript Title: "Impact of the preservation of residual kidney function on hemodialysis survival: results from the BISTRO trial"
Date of Completion: August 21, 2024
Disclosure Updated Date: August 21, 2024

ASN
American Society of Nephrology
JASN
Journal of the American Society of Nephrology
KIDNEY360
Accessing Our World From Every Angle
CJASN
Clinical Journal of the American Society of Nephrology

ASN Journal Disclosure Form

As per ASN journal policy, I have disclosed any financial relationships or commitments I have held in the past 36 months as included below. I have listed my Current Employer below to indicate there is a relationship requiring disclosure. If no relationship exists, my Current Employer is not listed.

F. Caskey reports the following:
Employer: University of Bristol; Research Funding: National Institute for Health Research; Kidney Research UK; PCORI.; and Advisory or Leadership Role: International Society of Nephrology (Treasurer, Honorary Secretary, Executive Committee member). Kidney Research UK. Unpaid.

I understand that the information above will be published within the journal article, if accepted, and that failure to comply and/or to accurately and completely report the potential financial conflicts of interest could lead to the following: 1) Prior to publication, article rejection, or 2) Post-publication, sanctions ranging from, but not limited to, issuing a correction, reporting the inaccurate information to the authors' institution, banning authors from submitting work to ASN journals for varying lengths of time, and/or retraction of the published work.

Name: Fergus Caskey
Manuscript ID: K360-2024-000151R2
Manuscript Title: Impact of the preservation of residual kidney function on hemodialysis survival: results from the BISTRO trial
Date of Completion: August 22, 2024
Disclosure Updated Date: April 23, 2024

ASN Journal Disclosure Form

As per ASN journal policy, I have disclosed any financial relationships or commitments I have held in the past 36 months as included below. I have listed my Current Employer below to indicate there is a relationship requiring disclosure. If no relationship exists, my Current Employer is not listed.

D. Coyle reports the following:
Employer: Cai-shen ltd

I understand that the information above will be published within the journal article, if accepted, and that failure to comply and/or to accurately and completely report the potential financial conflicts of interest could lead to the following: 1) Prior to publication, article rejection, or 2) Post-publication, sanctions ranging from, but not limited to, issuing a correction, reporting the inaccurate information to the authors' institution, banning authors from submitting work to ASN journals for varying lengths of time, and/or retraction of the published work.

Name: David Coyle
Manuscript ID: Manuscript ID K360-2024-000151R2
Manuscript Title: "Impact of the preservation of residual kidney function on hemodialysis survival: results from the BISTRO trial,"
Date of Completion: September 17, 2024
Disclosure Updated Date: August 22, 2024

ASN American Society of Nephrology JASN Journal of the American Society of Nephrology KIDNEY360 Accessing Our World From Every Angle CJASN Clinical Journal of the American Society of Nephrology

ASN Journal Disclosure Form

As per ASN journal policy, I have disclosed any financial relationships or commitments I have held in the past 36 months as included below. I have listed my Current Employer below to indicate there is a relationship requiring disclosure. If no relationship exists, my Current Employer is not listed.

I. Dasgupta reports the following:
Employer: University Hospitals Birmingham NHS Foundation Trust, UK; Research Funding: Sanofi and Baxter; Honoraria: GSK for being UK national leader of three GSK sponsored trials; GSK for advisory board meetings; AstraZaneca for advisory board meeting, Vifor for advisory board; Advisory or Leadership Role: GSK, Vifor, Sanofi, Medtronic; Speakers Bureau: AstraZeneca, GSK, Vifor; and Other Interests or Relationships: Expert Adviser to National Institute for Health and Care Excellence (NICE), UK; Chair, British and Irish Hypertension Society, Collaborative Research Standing Committee.

I understand that the information above will be published within the journal article, if accepted, and that failure to comply and/or to accurately and completely report the potential financial conflicts of interest could lead to the following: 1) Prior to publication, article rejection, or 2) Post-publication, sanctions ranging from, but not limited to, issuing a correction, reporting the inaccurate information to the authors' institution, banning authors from submitting work to ASN journals for varying lengths of time, and/or retraction of the published work.

Name: Indranil Dasgupta
Manuscript ID: K360-2024-000151R1
Manuscript Title: Impact of the preservation of residual kidney function on hemodialysis survival: results from the BISTRO trial.
Date of Completion: July 17, 2024
Disclosure Updated Date: July 17, 2024

ASN American Society of Nephrology
JASN Journal of the American Society of Nephrology
KIDNEY360 Accessing Our World From Every Angle
CJASN Clinical Journal of the American Society of Nephrology

ASN Journal Disclosure Form

As per ASN journal policy, I have disclosed any financial relationships or commitments I have held in the past 36 months as included below. I have listed my Current Employer below to indicate there is a relationship requiring disclosure. If no relationship exists, my Current Employer is not listed.

A. Davenport reports the following:
Consultancy: 1. Fresenius Medical Care - lecture on haemodiafiltration; 2. Nipro Corporation - lecture on alpha 1 macroglobulin; Ownership Interest: United utilities Ltd shares; Astra Zenica shares; Honoraria: 1. Fresenius Medical Company; 2. Nipro Corporation; and Advisory or Leadership Role: Advisory board -WAK Scientific advisory board; Leadership positions; European Dialysis & Transplant Association ERN committee ; past president - International Society for Hemodialysis,.

I understand that the information above will be published within the journal article, if accepted, and that failure to comply and/or to accurately and completely report the potential financial conflicts of interest could lead to the following: 1) Prior to publication, article rejection, or 2) Post-publication, sanctions ranging from, but not limited to, issuing a correction, reporting the inaccurate information to the authors' institution, banning authors from submitting work to ASN journals for varying lengths of time, and/or retraction of the published work.

Name: Andrew Davenport
Manuscript ID: K360-2024-000151R2
Manuscript Title: Impact of the preservation of residual kidney function on hemodialysis survival: results from the BISTRO trial
Date of Completion: August 25, 2024
Disclosure Updated Date: July 17, 2024

ASN
American Society of Nephrology
JASN
Journal of the American Society of Nephrology
KIDNEY360
Accessing Our World From Every Angle
CJASN
Clinical Journal of the American Society of Nephrology

ASN Journal Disclosure Form

As per ASN journal policy, I have disclosed any financial relationships or commitments I have held in the past 36 months as included below. I have listed my Current Employer below to indicate there is a relationship requiring disclosure. If no relationship exists, my Current Employer is not listed.

S. Davies reports the following:
Employer: Keele University; Consultancy: Ellen Medical; Honoraria: Baxter HealthCare; and Advisory or Leadership Role: International Society of Peritoneal Dialysis (Member, co-chair PDOPPS Committee); International Society of Nephrology (Kidney Failure Strategy); Vice-President EuroPD; Trustee Kidney Research UK (Chair of Research Strategy Committee).

I understand that the information above will be published within the journal article, if accepted, and that failure to comply and/or to accurately and completely report the potential financial conflicts of interest could lead to the following: 1) Prior to publication, article rejection, or 2) Post-publication, sanctions ranging from, but not limited to, issuing a correction, reporting the inaccurate information to the authors' institution, banning authors from submitting work to ASN journals for varying lengths of time, and/or retraction of the published work.

Name: Simon J. Davies
Manuscript ID: K360-2024-000151R1
Manuscript Title: Impact of the preservation of residual kidney function on hemodialysis survival: results from the BISTRO trial.
Date of Completion: July 17, 2024
Disclosure Updated Date: May 21, 2024

ASN American Society of Nephrology | JASN Journal of the American Society of Nephrology | KIDNEY360 Accessing Our World From Every Angle | CJASN Clinical Journal of the American Society of Nephrology

ASN Journal Disclosure Form

As per ASN journal policy, I have disclosed any financial relationships or commitments I have held in the past 36 months as included below. I have listed my Current Employer below to indicate there is a relationship requiring disclosure. If no relationship exists, my Current Employer is not listed.

K. Farrington reports the following:
Employer: Lister Renal Unit; Stevenage UK; University of Hertfordshire; Hatfield UK

I understand that the information above will be published within the journal article, if accepted, and that failure to comply and/or to accurately and completely report the potential financial conflicts of interest could lead to the following: 1) Prior to publication, article rejection, or 2) Post-publication, sanctions ranging from, but not limited to, issuing a correction, reporting the inaccurate information to the authors' institution, banning authors from submitting work to ASN journals for varying lengths of time, and/or retraction of the published work.

Name: Ken Farrington
Manuscript ID: K360-2024-000151R1
Manuscript Title: Impact of the preservation of residual kidney function on hemodialysis survival: results from the BISTRO trial
Date of Completion: July 17, 2024
Disclosure Updated Date: May 21, 2024

ASN American Society of Nephrology JASN Journal of the American Society of Nephrology KIDNEY360 Accessing Our World From Every Angle CJASN Clinical Journal of the American Society of Nephrology

ASN Journal Disclosure Form

As per ASN journal policy, I have disclosed any financial relationships or commitments I have held in the past 36 months as included below. I have listed my Current Employer below to indicate there is a relationship requiring disclosure. If no relationship exists, my Current Employer is not listed.

D. Keane reports the following:
Employer: University of Galway

I understand that the information above will be published within the journal article, if accepted, and that failure to comply and/or to accurately and completely report the potential financial conflicts of interest could lead to the following: 1) Prior to publication, article rejection, or 2) Post-publication, sanctions ranging from, but not limited to, issuing a correction, reporting the inaccurate information to the authors' institution, banning authors from submitting work to ASN journals for varying lengths of time, and/or retraction of the published work.

Name: David Francis Keane
Manuscript ID: K360-2024-000151R1
Manuscript Title: Impact of the preservation of residual kidney function on hemodialysis survival: results from the BISTRO trial
Date of Completion: July 17, 2024
Disclosure Updated Date: July 17, 2024

ASN Journal Disclosure Form

As per ASN journal policy, I have disclosed any financial relationships or commitments I have held in the past 36 months as included below. I have listed my Current Employer below to indicate there is a relationship requiring disclosure. If no relationship exists, my Current Employer is not listed.

E. Lindley reports the following:
Employer: Leeds Teaching Hospitals NHS Trust; and Consultancy: Diaverum AB (my husband designed the decision support system they use for anaemia management and occasionally makes adjustments to incorporate new products or practices).

I understand that the information above will be published within the journal article, if accepted, and that failure to comply and/or to accurately and completely report the potential financial conflicts of interest could lead to the following: 1) Prior to publication, article rejection, or 2) Post-publication, sanctions ranging from, but not limited to, issuing a correction, reporting the inaccurate information to the authors' institution, banning authors from submitting work to ASN journals for varying lengths of time, and/or retraction of the published work.

Name: Elizabeth J. Lindley
Manuscript ID: K360-2024-000151R1
Manuscript Title: Impact of the preservation of residual kidney function on hemodialysis survival: results from the BISTRO trial
Date of Completion: July 17, 2024
Disclosure Updated Date: July 17, 2024

ASN American Society of Nephrology | JASN Journal of the American Society of Nephrology | KIDNEY360 Accessing Our World From Every Angle | CJASN Clinical Journal of the American Society of Nephrology

ASN Journal Disclosure Form

As per ASN journal policy, I have disclosed any financial relationships or commitments I have held in the past 36 months as included below. I have listed my Current Employer below to indicate there is a relationship requiring disclosure. If no relationship exists, my Current Employer is not listed.

J. Macdonald reports the following:
Employer: Bangor University

I understand that the information above will be published within the journal article, if accepted, and that failure to comply and/or to accurately and completely report the potential financial conflicts of interest could lead to the following: 1) Prior to publication, article rejection, or 2) Post-publication, sanctions ranging from, but not limited to, issuing a correction, reporting the inaccurate information to the authors' institution, banning authors from submitting work to ASN journals for varying lengths of time, and/or retraction of the published work.

Name: Jamie Hugo Macdonald
Manuscript ID: K360-2024-000151R1
Manuscript Title: Impact of the preservation of residual kidney function on hemodialysis survival: results from the BISTRO trial
Date of Completion: July 17, 2024
Disclosure Updated Date: May 10, 2024

ASN American Society of Nephrology | JASN Journal of the American Society of Nephrology | KIDNEY360 Accessing Our World From Every Angle | CJASN Clinical Journal of the American Society of Nephrology

ASN Journal Disclosure Form

As per ASN journal policy, I have disclosed any financial relationships or commitments I have held in the past 36 months as included below. I have listed my Current Employer below to indicate there is a relationship requiring disclosure. If no relationship exists, my Current Employer is not listed.

S. Mitra reports the following:
Research Funding: Fresenius, Invizius; Honoraria: Vifor Pharma, GSK, Astra Zeneca; Advisory or Leadership Role: Invizius; and Speakers Bureau: Astra Zeneca.

I understand that the information above will be published within the journal article, if accepted, and that failure to comply and/or to accurately and completely report the potential financial conflicts of interest could lead to the following: 1) Prior to publication, article rejection, or 2) Post-publication, sanctions ranging from, but not limited to, issuing a correction, reporting the inaccurate information to the authors' institution, banning authors from submitting work to ASN journals for varying lengths of time, and/or retraction of the published work.

Name: Sandip Mitra
Manuscript ID: K360-2024-000151R1
Manuscript Title: Impact of the preservation of residual kidney function on hemodialysis survival: results from the BISTRO trial
Date of Completion: July 17, 2024
Disclosure Updated Date: May 20, 2024

ASN Journal Disclosure Form

As per ASN journal policy, I have disclosed any financial relationships or commitments I have held in the past 36 months as included below. I have listed my Current Employer below to indicate there is a relationship requiring disclosure. If no relationship exists, my Current Employer is not listed.

P. Ormandy reports the following:
Employer: University of Salford; and Advisory or Leadership Role: Association of Nephrology Nursing UK ; Kidney Patient Involvement Network, Kidney Information Network.

I understand that the information above will be published within the journal article, if accepted, and that failure to comply and/or to accurately and completely report the potential financial conflicts of interest could lead to the following: 1) Prior to publication, article rejection, or 2) Post-publication, sanctions ranging from, but not limited to, issuing a correction, reporting the inaccurate information to the authors' institution, banning authors from submitting work to ASN journals for varying lengths of time, and/or retraction of the published work.

Name: Paula Ormandy
Manuscript ID: K360-2024-000151R1
Manuscript Title: Impact of the preservation of residual kidney function on hemodialysis survival: results from the BISTRO trial
Date of Completion: July 17, 2024
Disclosure Updated Date: July 17, 2024

ASN JASN Journal of the American Society of Nephrology KIDNEY360 Accessing Our World From Every Angle CJASN Clinical Journal of the American Society of Nephrology

ASN Journal Disclosure Form

As per ASN journal policy, I have disclosed any financial relationships or commitments I have held in the past 36 months as included below. I have listed my Current Employer below to indicate there is a relationship requiring disclosure. If no relationship exists, my Current Employer is not listed.

J. Sim reports the following:
Employer: Keele University

I understand that the information above will be published within the journal article, if accepted, and that failure to comply and/or to accurately and completely report the potential financial conflicts of interest could lead to the following: 1) Prior to publication, article rejection, or 2) Post-publication, sanctions ranging from, but not limited to, issuing a correction, reporting the inaccurate information to the authors' institution, banning authors from submitting work to ASN journals for varying lengths of time, and/or retraction of the published work.

Name: Julius Sim
Manuscript ID: K360-2024-000151R2
Manuscript Title: IImpact of the preservation of residual kidney function on hemodialysis survival: results from the BISTRO trial
Date of Completion: August 21, 2024
Disclosure Updated Date: August 21, 2024

ASN
American Society of Nephrology
JASN
Journal of the American Society of Nephrology
KIDNEY360
Accessing Our World From Every Angle
CJASN
Clinical Journal of the American Society of Nephrology

ASN Journal Disclosure Form

As per ASN journal policy, I have disclosed any financial relationships or commitments I have held in the past 36 months as included below. I have listed my Current Employer below to indicate there is a relationship requiring disclosure. If no relationship exists, my Current Employer is not listed.

I. Solis-Trapala has nothing to disclose.

I understand that the information above will be published within the journal article, if accepted, and that failure to comply and/or to accurately and completely report the potential financial conflicts of interest could lead to the following: 1) Prior to publication, article rejection, or 2) Post-publication, sanctions ranging from, but not limited to, issuing a correction, reporting the inaccurate information to the authors' institution, banning authors from submitting work to ASN journals for varying lengths of time, and/or retraction of the published work.

Name: Ivonne Solis-Trapala
Manuscript ID: K360-2024-000151R1
Manuscript Title: Impact of the preservation of residual kidney function on hemodialysis survival: results from the BISTRO trial
Date of Completion: July 20, 2024
Disclosure Updated Date: July 20, 2024

ASN
American Society of Nephrology
JASN
Journal of the American Society of Nephrology
KIDNEY360
Accessing Our World From Every Angle
CJASN
Clinical Journal of the American Society of Nephrology

ASN Journal Disclosure Form

As per ASN journal policy, I have disclosed any financial relationships or commitments I have held in the past 36 months as included below. I have listed my Current Employer below to indicate there is a relationship requiring disclosure. If no relationship exists, my Current Employer is not listed.

M. Wilkie reports the following:
Employer: Sheffield Teaching Hospitals;; Consultancy: Triomed; Research Funding: Baxter; Honoraria: Baxter, Fresenius; Speakers Bureau: Baxter; and Other Interests or Relationships: Ineternational Society for Peritoneal Dialysis.

I understand that the information above will be published within the journal article, if accepted, and that failure to comply and/or to accurately and completely report the potential financial conflicts of interest could lead to the following: 1) Prior to publication, article rejection, or 2) Post-publication, sanctions ranging from, but not limited to, issuing a correction, reporting the inaccurate information to the authors' institution, banning authors from submitting work to ASN journals for varying lengths of time, and/or retraction of the published work.

Name: Martin E. Wilkie
Manuscript ID: K360-2024-000151R2
Manuscript Title: Impact of the preservation of residual kidney function on hemodialysis survival: results from the BISTRO trial
Date of Completion: August 20, 2024
Disclosure Updated Date: September 16, 2023
